# Characterization and *in ovo* vascularization of a 3D-printed hydroxyapatite scaffold with different extracellular matrix coatings under perfusion culture

**DOI:** 10.1242/bio.034488

**Published:** 2018-10-19

**Authors:** Floriana Burgio, Natalie Rimmer, Uwe Pieles, Johanna Buschmann, Marina Beaufils-Hugot

**Affiliations:** 1 School of Life Sciences, Institute for Chemistry and Bioanalytics (ICB), Gründenstrasse 40, CH-4132 Basel, Switzerland; 2 University Hospital Zürich (USZ), Plastic Surgery and Hand Surgery, Sternwartstrasse 14, CH-8091 Zürich, Switzerland

**Keywords:** Perfusion culture, Ceramic, Elastic modulus, Osteogenesis, Angiogenesis, CAM assay

## Abstract

For the fabrication of appropriate bone tissue-engineered constructs several prerequisites should be fulfilled. They should offer long-term stability, allow proper cell attachment and proliferation and furthermore be osteoinductive and easy to be vascularized. Having these requirements as background, we fabricated a novel porous 3D-printed hydroxyapatite (HA) scaffold and treated it with oxygen plasma (OPT). MG-63 pre-osteoblast-seeded bone constructs allowed good cell attachment and proliferation, which was even better when cultivated in a perfusion flow bioreactor. Moreover, the deposition of extracellular matrix (ECM) on the otherwise inorganic surface changed the mechanical properties in a favourable manner: elasticity increased from 42.95±1.09 to 91.9±5.1 MPa (assessed by nanoindentation). Compared to static conditions, osteogenic differentiation was enhanced in the bioreactor, with upregulation of ALP, collagen I and osteocalcin gene expression. In parallel experiments, primary human bone marrow mesenchymal stromal cells (hBMSCs) were used and findings under dynamic conditions were similar; with a higher commitment towards osteoblasts compared to static conditions. In addition, angiogenic markers CD31, eNOS and VEGF were upregulated, especially when osteogenic medium was used rather than proliferative medium. To compare differently fabricated ECMs in terms of vascularization, decellularized constructs were tested in the chorioallantoic membrane (CAM) assay with subsequent assessment of the functional perfusion capacity by MRI in the living chick embryo. Here, vascularization induced by ECM from osteogenic medium led to a vessel distribution more homogenous throughout the construct, while ECM from proliferative medium enhanced vessel density at the interface and, to a lower extent, at the middle and top. We conclude that dynamic cultivation of a novel porous OPT HA scaffold with hBMSCs in osteogenic medium and subsequent decellularization provides a promising off-the-shelf bone tissue-engineered construct.

## Abstract (precis)

**Summary:** Depending on the culture media, ECM coating of 3D-printed hydroxyapatite evokes different functional vascularization as assessed by CAM assay *in ovo*. ECM coating enhances vascularization compared to ECM-free hydroxyapatite.

## INTRODUCTION

In maxillofacial and orthopaedic surgery, repair and regeneration of bone defects caused by trauma, tumour excision or infection is daily business and is a central clinical goal. To treat bone tissue loss, the development of novel orthopaedic strategies based on tissue engineering approaches has progressed considerably over the last four decades (Langer et al., 1990; Venkatesan and Kim, 2010; Boland, 2017; Forrestal et al., 2017; Hosseinpour et al., 2017; Khakestani et al., 2017; Kim et al*.*, 2017; Janko et al., 2018).

In particular, bone tissue engineering aims to regenerate damaged tissues by combining osteogenic cells with highly porous biomaterials, which act as templates for tissue regeneration, and subsequent osteointegration (Amini et al., 2012; Boland, 2017). Among the different kinds of biomaterials (polymers, ceramics and composite materials), calcium phosphate ceramics are widely investigated thanks to excellent biocompatibility, bioactivity and osteoconductivity. In particular, hydroxyapatite (HA)-based scaffolds (Lee et al., 2013; Ha et al*.*, 2015; Dang et al., 2016) are of considerable interest, since HA is the major inorganic component of natural bone (Nandi et al., 2010). One major drawback of pure HA scaffolds, however, is their brittleness (Shi et al., 2014; Owen et al., 2017), which has been overcome by various approaches, such as composites with polymers (Roeder et al., 2008; Cox et al*.*, 2015; Zeng et al., 2018) or by the deposition of extracellular matrix (Sadr et al., 2012), which increases the elasticity of the materials.

Besides gas or chemical foaming (Kucharska et al., 2012), salt leaching (Cao and Kuboyama, 2010; Stoppato et al., 2013) or thermally induced phase separation (Hutmacher, 2000), 3D printing is a suitable and well established technique to produce porous HA biomaterials, as it provides tailored templates that are able to enhance cell adhesion, proliferation, differentiation and neovascularization (Leukers et al., 2005; Bose et al., 2013, 2018; Forrestal et al., 2017; Shaunak et al., 2017; Zhang et al., 2017a,b; Zeng et al., 2018). 3D printing of HA has been reported to be successful for craniomaxiofacial regeneration (Gaviria et al., 2017), among others (Heller et al., 2016).

To better reproduce an *in vivo*-like environment, the use of 3D cell-seeded scaffolds cultivated in a perfusion bioreactor system has been shown (i) to improve cell seeding efficiency (Gardel et al., 2014), (ii) to maintain a uniform distribution of viable cells throughout scaffolds (Lee et al., 2017) and (iii) to overcome the limited mass exchange of nutrients and oxygen observed under static conditions (Bancroft et al., 2002; Wendt et al., 2003; Kashte et al., 2017). Finally, bone tissue engineered scaffolds cultured in a perfusion bioreactor showed a better *in vivo* performance compared to statically cultivated scaffolds (Yeatts et al., 2014).

Hence, the hypotheses of our study were that:

- ECM deposition enhances elastic properties of a 3D-printed HA scaffold,
- perfusion culture improves cell infiltration into the macro- and micro-pores of the scaffold and
- ECM deposition enhances vascularization of 3D-printed HA scaffold.

## RESULTS

### Scaffold architecture, microstructure and mechanical properties

**Fig. 1.**
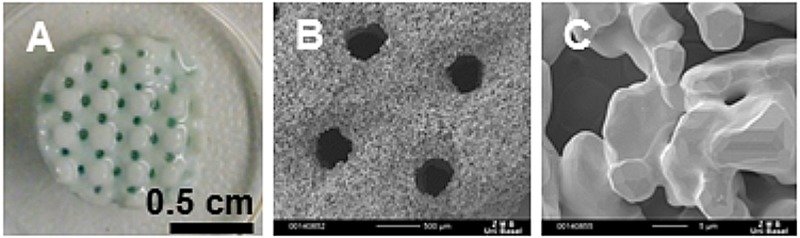
**3D printed hydroxyapatite scaffold with defined macroporosity.** Scale bars: 0.5 cm (A), 500 µm (B) and 5 µm (C).

Macroscopic and SEM images of porous HA scaffolds produced by a 3D printing method and sintered at 1425°C are shown in Fig. 1. In addition to the printed, geometric macroporous structure with pores ranging from 300 to 600 µm (Fig. 1A,B), a microporous structure, with pores of 10–15 µm, was observed inside the material at higher magnification (Fig. 1C). Upon deposition of ECM by hBMSCs, the elastic modulus, as assessed by nanoindentation, increased for the non-devitalized scaffold from 42.95±1.09 (cell-free) to 91.9±5.1 MPa (cell-seeded).

Standard compression tests resulted in an elastic modulus of the bulk scaffold of 14.2±7.9 MPa (cell-free) and 19.3±2.9 MPa (cell-seeded).

### Cell seeded 3D-printed HA scaffolds: static versus dynamic culture

**Fig. 2.**
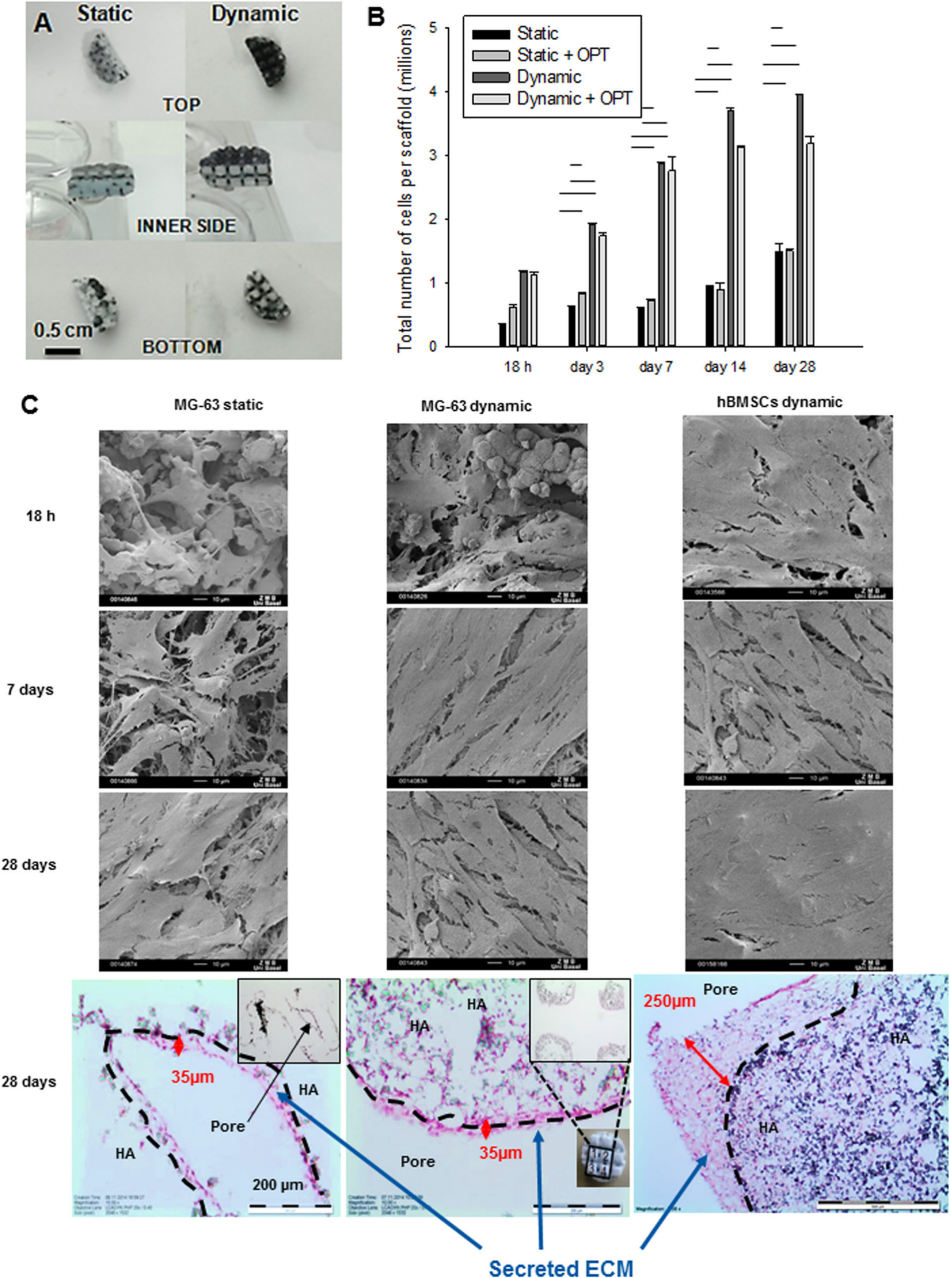
**Cell attachment and proliferation on 3D-printed HA scaffold under static versus dynamic conditions.** (A) MTT staining after 18 hours of cell seeding under static (left) and dynamic (right) conditions. (B) Cell number based on DNA content of cells seeded on untreated or oxygen-plasma treated (OPT) scaffolds for up to 28 days of culture under static or dynamic conditions. (C) SEM images of MG-63 cells cultivated on 3D-printed HA scaffolds after 18 h, 7 and 28 days of culture in static (left column) and dynamic conditions (middle column) and of hBMSCs cultivated under dynamic conditions (right column), scale bar: 10 μm. Lower panels show histological H&E stained sections of corresponding cell-seeded scaffolds after 28 days (scale bars: 500 μm).

The proliferation of MG-63 osteoblast-like cells seeded on 3D-printed porous HA scaffolds after 18 h, 3, 7, 14 and 28 days of culture under static (24-well plate) and dynamic (perfusion bioreactor) conditions was evaluated. An MTT assay was used as a qualitative method to visualize cell viability. After 18 h of cell seeding under static conditions, cell distribution was not homogeneous, and only a few cells were present at the bottom part of the scaffold, as shown in Fig. 2A. In contrast, under dynamic conditions, there were more cells and cell distribution was more homogeneous, with cells covering the whole surface of the scaffold. Based on the analysis of the DNA content, cell number was assessed with or without OPT of the HA scaffolds (Fig. 2B).

This cell quantification was performed 18 h, 3, 7, 14 and 28 days after seeding, and static and dynamic conditions were compared. In static cultures, the number of MG-63 cells in the constructs did not significantly increase after 28 days of culture; even after hydrophilic surface modification (i.e. with OPT). In contrast, under perfusion culture, a three- to fourfold increase in cell number was observed from 18 h to 28 days of culture. Moreover, the cell number reached a plateau after 7 (threefold) and 14 (fourfold) days of culture, respectively, with or without OPT. Cell attachment and spreading were assessed by SEM analysis (Fig. 2C). MG-63 cells grown for 28 days under static conditions (Fig. 2C, first column) adhered and grew on the HA matrix but left some uncovered areas. In contrast, the osteosarcoma cells grown under perfusion culture (Fig. 2C, second column) showed a dense cell multilayer, with a more homogeneous cell distribution, already 7 days after cell seeding.

In similar perfusion culture conditions, hBMSCs exhibited even denser cell coverage, already 18 h after cell seeding (Fig. 2C, third column). After 28 days of culture a homogenous and continuous coating made of cells but also of secreted ECM was observed in the histological sections (Fig. 2C, third row).

Histological analysis was performed to analyse tissue formation and cell invasion into the material under static and dynamic conditions after 28 days of culture. Under static conditions, MG-63 cells generated a thin tissue layer (35 µm) at the surface of the pores of HA scaffolds, with limited evidence of cell ingrowth inside the material (Fig. 2C, third row on the left). Under dynamic conditions, MG-63 cells generated a similar tissue at the surface of the 3D-printed ceramic, but was denser in terms of cells coating, now also invading the inner part of the biomaterial (Fig. 2C, third row in the middle). When hBMSC cells were cultivated with perfusion flow under the same conditions, the tissue thickness reached 250 µm (sevenfold increase) after 28 days and cell infiltration into the microstructure of the biomaterial was even denser in comparison to MG-63 cells (Fig. 2C, third row on the right).

### Osteogenic differentiation of MG-63 cells and hBMSC

#### MG-63 cells

**Fig. 3.**
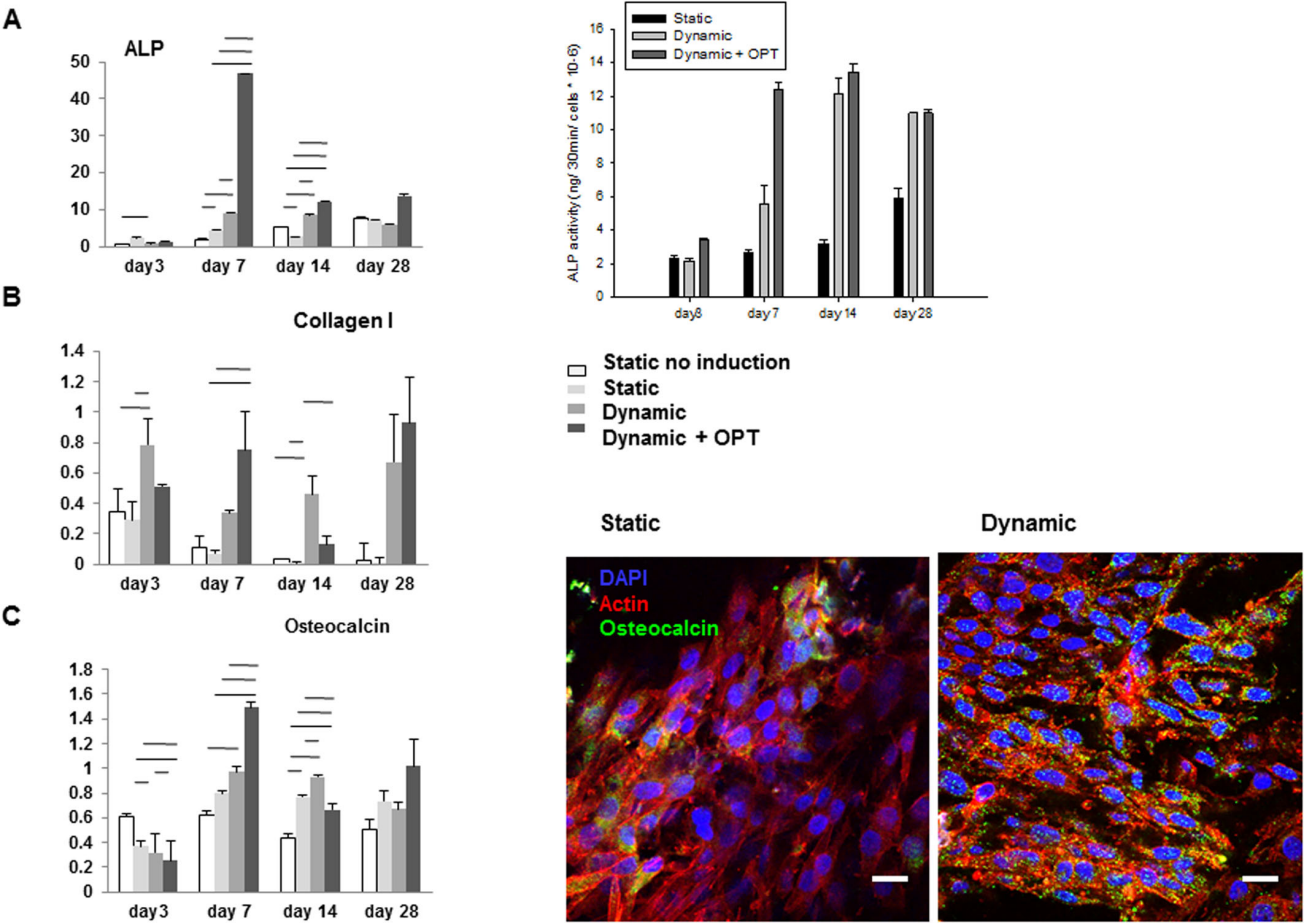
**Differentiation of MG-63 human osteoblast-like cells cultured on 3D-printed porous HA scaffolds under static versus dynamic conditions.** Real-time PCR analysis of (A) ALP (left) and ALP activity (right); (B) Collagen I and (C) Osteocalcin expressed by MG-63 cells under static and dynamic conditions after 3, 7, 14 and 28 days of culture, and corresponding immunofluorescence staining after 28 days of culture under static and dynamic conditions. Scale bars: 20 µm.

The gene expression of alkaline phosphatase (ALP), collagen I and osteocalcin was analyzed by PCR. Markers of osteoblastic differentiation, namely ALP and osteocalcin, showed a peak of gene expression at day 7 under dynamic (+OPT) conditions and higher expression in dynamic conditions as compared to static culture (Fig. 3). Collagen I mRNA expression was much higher with perfusion flow than in static conditions after 28 days of culture. Higher expression of osteocalcin by MG-63 cells in dynamic perfusion culture was qualitatively confirmed at the protein level at 28 days of culture by immunofluorescence. More functionally, under perfusion flow, ALP activity by MG-63 increased from day 3 to day 14 to reach a peak after 2 weeks of culture. ALP activity of MG-63 cells cultured on oxygen-plasma pre-treated scaffolds in dynamic conditions reached the plateau value at day 7.

#### hBMSCs

**Fig. 4.**
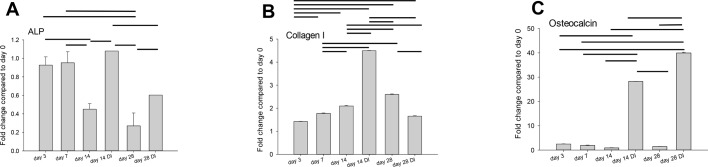
**Real-time PCR analysis of bone-associated genes expressed by hBMSC cells cultured in dynamic condition on 3D-porous HA scaffolds.** (A) ALP. (B) Collagen I. (C) Osteocalcin. Cultured in proliferation medium or differentiation medium (DI).

hBMSCs cultured under perfusion exhibited strong osteoblastic differentiation with increased gene expression of markers such as ALP, collagen I and osteocalcin when compared to undifferentiated hBMSCs (Fig. 4).

### Perfusion capacities of decellularized scaffolds in the CAM assay

**Fig. 5.**
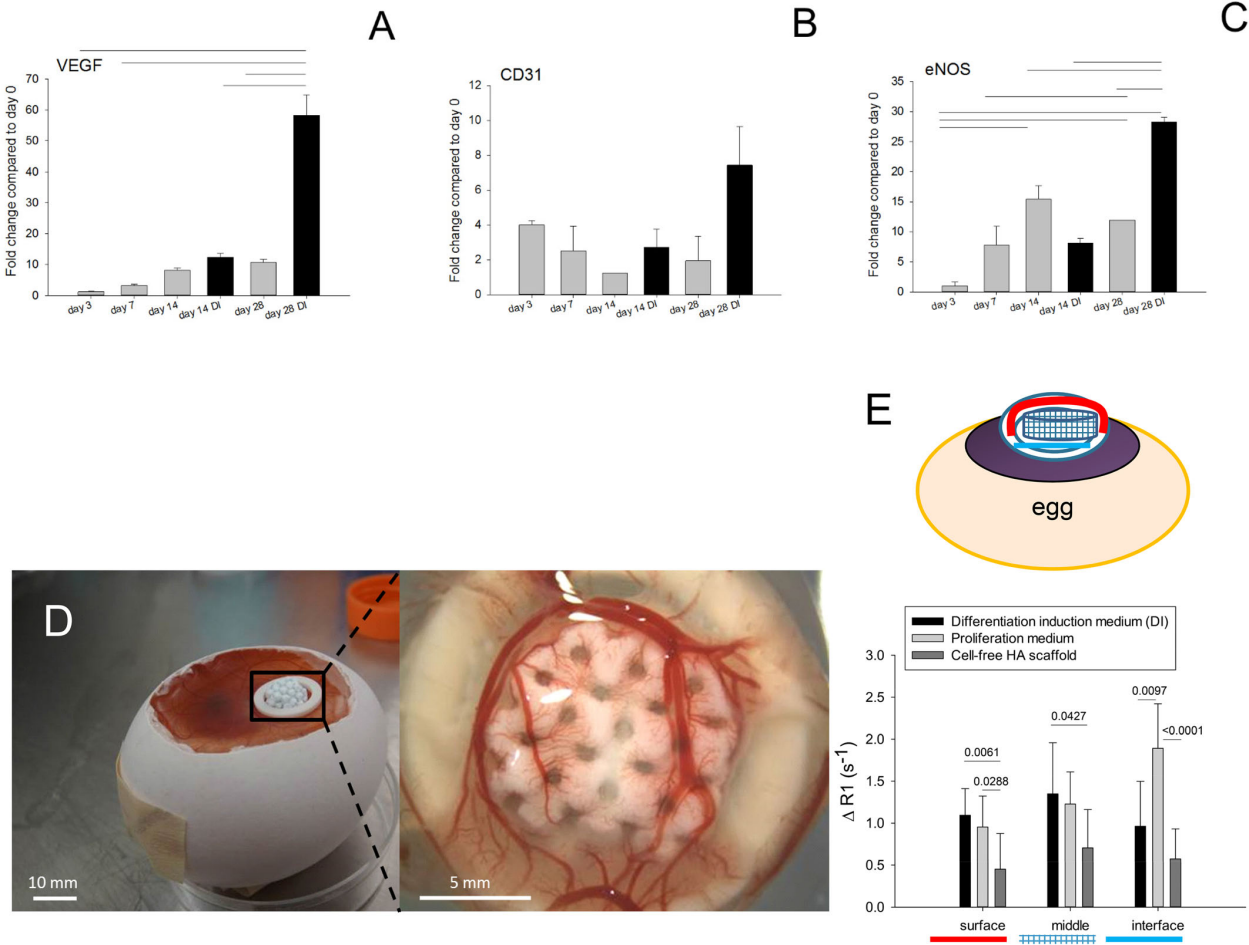
**Real-time PCR analysis angiogenesis-related genes expressed by hBMSC cells cultured under dynamic condition on 3D-porous HA scaffolds and typical angiogenesis assay.** The CAM assay allows *in ovo* vascularistion of biomaterials planted on the surface. (A–C) Manifold gene expression of (A) VEGF, (B) CD31and (C) eNOS cultured in proliferation medium (grey bars) or osteogenic differentiation medium (DI, black bars). (D) Windowed egg at incubation day ID7 when HA scaffolds were onplanted (left) and vascularized HA scaffold coated with ECM from osteoinductive medium at ID14 after 1 week on the CAM (right). (E) Relative relaxation rates as assessed in the MRI of HA scaffolds decorated with ECM produced by cells cultivated in osteogenic medium with differentiation induction (black bars) or in proliferative medium (light grey bars) and of uncoated HA scaffolds (dark grey bars). For the precise composition of the two culture media, see Materials and Methods section. A scheme (on top) represents the different regions of the scaffold (surface, middle and interface) of an egg.

Angiogenic markers genes, such as VEGF, CD31 and eNOS exhibited significantly higher gene expression levels (VEGF, *P*<0.0001; CD31, *P*=0.0078; eNOS, *P*<0.0001) with differentiation medium (DI) than with proliferative medium (Fig. 5A–C).

MRI of the living chicken embryos with onplanted decellularized scaffolds as well as uncoated HA scaffolds in the windowed egg (Fig. 5D) was performed to analyse perfusion capacity of the constructs (functional vessels). Although only at the interface, statistically significant differences were found between osteogenic medium and proliferation medium (Fig. 5E). The two different ECMs attracted vessels to a significantly higher extent into the construct compared to cell-free HA.

## DISCUSSION

In this study, we characterized a novel porous 3D HA scaffold aimed at bone tissue engineering (Fig. 1). First, the 3D HA scaffold was oxygen-plasma treated (+OPT) and seeded with MG-63 pre-osteoblasts. Cell-seeded scaffolds±OPT were either cultivated under static conditions or in a perfusion flow bioreactor under dynamic conditions. Compared to static cultivation, dynamic cultivation improved cell attachment and proliferation of MG-63 cells significantly (Fig. 2). Moreover, dynamic cultivation increased mRNA expression of typical osteogenic marker genes like ALP, collagen I or osteocalcin compared to static cultivation (Fig. 3). 3D HA scaffolds especially, when treated with OPT, triggered the desired differentiation in MG-63 pre-osteoblasts as shown by a significant increase of ALP expression on day 7. Of note, ECM deposition also improved the mechanical properties of the scaffold with an increased Young's modulus compared to cell-free scaffolds.

### Cell seeding and differentiation

For the initial tests, MG-63 cells served well as an *in vitro* model cell line. However, due to their low capacity for calcium deposition and their lack for osteoblastic function, their different proliferation rate, ALP activity and ECM formation, human primary bone-marrow derived mesenchymal stem cells (hBMSCs) were used for further optimization of the cell-enhanced biomaterial. Hence, human BMSC-seeded OPT 3D HA scaffolds were cultivated under dynamic conditions. In order to support osteogenic differentiation, osteoinductive medium (DI, differentiation induction) was used and compared to proliferative medium. As expected, osteogenic marker genes were expressed significantly more in osteogenic medium compared to proliferative medium (Fig. 4). In addition, angiogenic marker genes like VEGF, CD31 and eNOS were significantly increased in DI medium (Fig. 5A­–C). As angiogenesis always precedes osteogenesis and only both processes together ensure true bone formation (Scherberich et al., 2010; Wang et al., 2010; Wu et al*.*, 2017), we tested the two scaffolds after decellularization with regards to vascularization. In other words, the two different ECMs, produced either by hBMSCs in proliferative or osteogenic culture medium, respectively, were compared with regards to their angiogenic potential in the CAM assay (Fig. 5D). Although not significantly different, there was a trend for higher vessel density and perfusion capacity in the upper part of the vascularized scaffolds evoked by the ECM fabricated in osteogenic medium (Fig. 5E). In contrast, ECM coating from cultures in proliferative medium had a higher perfusion capacity at the interface than at the middle or top of the scaffolds.

### Static versus dynamic cultivation

It has been shown in several studies that dynamic cultivation of stem cells enhances osteogenesis. For example, Silva and colleagues reported the beneficial effect on adipose-derived stromal cells (ASCs) if seeded on a bioactive glass foam and cultivated in a perfusion bioreactor in terms of osteogenesis (Silva et al., 2014). Moreover, enhancement of human ASCs' proliferation and differentiation towards osteoblasts was also confirmed when cells were seeded on a blend of corn starch and polycaprolactone (Rodrigues et al., 2012). For the hBMSCs used in our study, it was also reported that osteogenesis is supported by perfusion flow conditions – even for a short time of perfusion such as 2 h (Filipowska et al., 2016). Hence, our findings stand in accordance with results obtained from many different systems including mesenchymal stromal cells seeded on a scaffold material and exposed to dynamic culture conditions in form of perfusion flow. Mechanistic aspects beneath the finding that perfusion flow may trigger osteogenesis lie in the mechanobiology of cells; shear stress deforming the cells is a typical trigger (McCoy and O'Brien, 2010; Yourek et al., 2010; Bodle et al., 2011; Stavenschi et al., 2017) as well as enhanced cell-to-cell communication due to closer proximity of the cells evoked by the perfusion (Tang et al., 2010). Moreover, as clearly shown in our study, perfusion regimen leads to a dense ECM deposition, especially for hBMSCs, and thus increases the scaffold's elastic modulus twofold. Different elasticity of the scaffold encountered by the cells leads to an enhanced osteogenesis (Engler et al., 2006; Zhang et al., 2017a,b). In summary, dynamic cultivation evokes changes on different levels – directly by the deformation of the cells exposed to shear stress, but also indirectly, by enhancing proliferation and increasing cell density and in doing so affecting cell-to-cell interactions, and finally by changing the ECM depo- and compositions and, thereby, modifying the mechanical properties of the scaffold material during the time of the experiment.

### Angiogenic potential

Angiogenesis is a main issue in bone tissue engineering because it has been shown that many bone grafts constructed *in vitro* do not perform satisfactorily *in vivo* – necrotic parts caused by insufficient vascularization may be faced. Many attempts have been undertaken to overcome this problem (Laschke et al., 2007; Scherberich et al., 2010; Helmrich et al., 2013). Strategies like VEGF application, arterio-venous loop construction (Manasseri et al., 2007), micro-tissue based bottom-up approaches (Declercq et al., 2013) or implementation of vasculogenic cells (Amini et al., 2016) have been undertaken. Here, we focused on the effect of ECM deposition on vascularization when the decellularized ECM-coated HA was planted onto the CAM. The effect of ECM deposition on synthetic polymers has been shown to overcome limited biological functionality (Sadr et al., 2012; Bourgine et al., 2017). The CAM assay is an easy and cheap *in vivo* (*in ovo*) assay, where the perfusion capacity in biomaterials can be easily assessed by MRI (Kivrak Pfiffner et al., 2014).

In order to answer whether or not two differently fabricated ECMs deposited by BMSCs that have been cultivated under perfusion, but in two different culture media (either in osteogenic or in proliferative medium), would have any impact on the functional vascularization of those constructs compared to cell-free scaffolds, we cultivated the decellularized constructs for 1 week on the CAM and assessed the relative relaxation rates in three different regions of the construct by MRI (at the interface, in the middle and at the surface). mRNA expression levels of VEGF and eNOS were significantly higher in hBMSCs (around 5- and 2.5-fold, respectively) when cultivated for 4 weeks in osteogenic medium rather than in proliferation medium (Fig. 5A­–C). Nevertheless, these changes in gene expression level did not significantly impact the osteogenic medium-related ECM containing this information. In contrast, at the interface to the CAM, there was a higher perfusion capacity found for ECM coating from proliferative rather than osteogenic medium. Compared to cell-free scaffolds, however, the ECM-coated scaffolds (from both media) attracted significantly more vessels from the CAM into the 3D-printed HA and this resulted in a significantly higher perfusion capacity (Fig. 5D,E). Although the constructs were completely decellularized, the two ECMs obtained differed in their angiogenic potential compared to the cell-free scaffolds, probably caused by a higher elastic modulus of the surface as determined by nanoindentation, facilitating the vessels to grow into the ECM-coated pores. Interestingly, reports on improved biocompatibility and increased osteoblastic differentiation of newly seeded pre-osteoblasts on ECM-coated scaffolds have reported upregulation of typical osteoblastic genes (Kim et al., 2018). However, the influence of ECM-coating by hBMSCs with an upregulation of both typical osteogenic and angiogenic genes, respectively, on *in vivo* functional performance (perfusion capacity of functional vessels) have not been reported so far. The observed effect is interesting in terms of bone grafts inducing vessel-gradients, offering osteochondral interface as a potential application (Camarero-Espinosa and Cooper-White, 2017). In summary, 3D-printed HA bone grafts can be instructed to attract vessels in different ways by decoration with specifically generated ECMs, and ECM coating leads to a higher *in ovo* perfusion capacity compared to cell-free HA scaffolds.

### Limitations

Although we applied the two ECM-coated and ECM-free scaffolds *in vivo* and assessed functional perfusion capacity *in ovo*, the CAM assay is restricted by a time window of 7 days where biomaterials can be vascularized. This is a comparatively short period when compared to other pre-clinical animal models that allow study of vascularization over a longer time. Although we found a different vascularization pattern for the two ECMs only at the interface, ECM coating enhanced the perfusion capacity *in ovo* significantly when compared to ECM-free analogues. Also, we only tested two different culture media and their corresponding ECMs, which might be interesting when enlarged – with the addition of different types of media to test if further vascularization patterns may be realized.

## CONCLUSIONS

We conclude that cultivation of human BMSCs on an OPT 3D HA scaffold intended for bone tissue engineering is favoured in dynamic conditions over static conditions, because osteogenesis is enhanced and triggered *in vitro*. Moreover, dynamic cultivation in osteogenic medium rather than proliferative medium upregulates typical angiogenic marker genes and may help to direct succeeding *in vivo* vascularization of the decellularized ECM-coated scaffold towards a fully vascularized and functional graft – as shown by our experiments in the CAM assay.

## MATERIALS AND METHODS

**Fig. 6.**
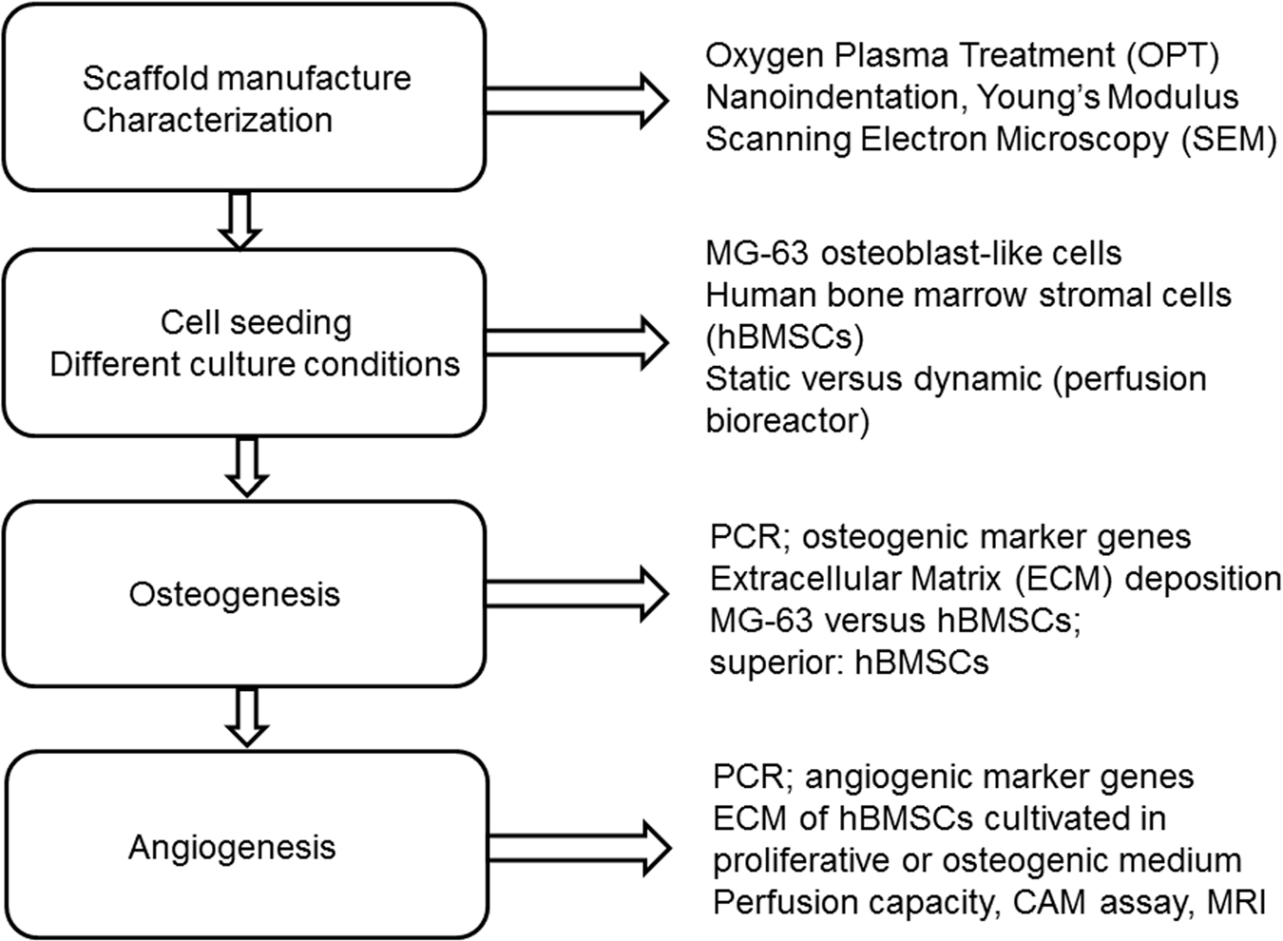
**Overview of experimental design.** Steps to tailor the 3D-printed ceramic scaffold (left) and methods used, as well as the parameters assessed at each step (right).

An overview of the experimental design is given in Fig. 6, including experimental steps to realize a scaffold with tailored osteoinductive and angiogenic properties.

### Preparation of the HA scaffold by 3D printing

Scaffolds were produced at the Institut für Medizinal und Analysetechnologien (IMA) of the Fachhochschule Nordwestschweiz (FHNW, Muttenz, Switzerland). Discs (10 mm diameter, 4 mm thick) were produced from HA powder (Medicoat AG, Mägenwil, Switzerland, MF/09-4964-03) with a 3D-printing system (3D-Printer Z-510, Z-Corporation). During the printing process, 10 wt. % citric acid and 15 wt. % phosphoric acid were added to the powder as a binder. In order to consolidate the specimens, sintering of ceramic materials was performed at 1425°C for 2 h (Carbolite RHF 1500). Scaffolds had an internal porosity of 61%, which was assessed by Archimedes' principle (Taylor et al., 1999), and an internal pore dimension ranging from 300 to 600 μm (macropores) and from 10 to 15 µm (micropores). The pore size was assessed in SEM images, with *n*=50.

### Cell culture and scaffold seeding

MG-63 human osteoblast-like cells are osteosarcoma cells and were a kind gift from René Prétôt [Laboratory of Toxicology, Institut für Chemie und Bioanalytik (ICB), Fachhochschule, Muttenz, Switzerland]. MG-63 cells were cultured in Dulbecco's Modified Eagle Medium high glucose (DMEM; Sigma-Aldrich, D6429) containing 1% penicillin/streptomycin/glutamine (Life Technologies, Invitrogen, 10378-016) supplemented with 10% fetal bovine serum (FBS; Gibco), 1% MEM non-essential amino acids (Life Technologies, 11140-035) and kept at37°C and 5% CO_2_.

hBMSC primary cells were obtained from eight healthy donors aged 36–54 years from different marrow cavities during routine orthopaedic surgery in accordance with the local ethical committee (University Hospital Basel) as previously described (Braccini et al., 2005) and cultured in α-Modified Eagle's Medium containing 10% FBS, 100 mM HEPES buffer solution, 1 mM sodium pyruvate, 100 U/ml penicillin, 100 µg/ml streptomycin and 292 µg/ml L-glutamine (Gibco), 100 nM dexamethasone (Sigma-Aldrich), 100 µM ascorbic acid 2 phosphate and 5 ng/ml fibroblast growth factor-2 (FGF-2; R&D Systems). The media were changed twice a week. Upon 80-90% cell confluency, cells were detached (0.5 g/l Trypsin/0.2 g/l EDTA, Invitrogen), and cell number and viability were assessed in a Neubauer chamber with trypan-blue dye exclusion test. Discs of porous HA scaffolds (10 mm diameter and 4 mm thick) were sterilized by oxygen-plasma treatment (OPT) for 2 min [Harrick Plasma; Expanded Plasma Cleaner (PDC-002)] according to the manufacturer's instructions. Scaffolds without OPT were sterilized by autoclaving at 121°C and 15 psi for 15 min. The two sterilization processes were necessary because OPT was, besides the surface treatment, also a sterilization process, while for the non-OPT treated samples, another sterilization process (autoclaving) had to be used.

### Static cultivation

Scaffolds used for static conditions were pre-soaked in proliferative culture medium overnight at 37°C and 5% CO_2_. Then they were placed one per well in an agarose-coated 24-multiwell plate to avoid any plastic cell adherence. 1×10^6^ cells were slowly dispersed over the top of each scaffold within a small volume (50 µl) of maintenance medium. After seeding, the cell constructs were incubated for 2.5 h to allow initial cell adhesion, before the addition of 1.5 ml of complete medium per well.

### Dynamic cultivation (perfusion flow)

For 3D culture, HA scaffolds were seeded by 1×10^6^ MG-63 or hBMSC cells and placed into a perfusion bioreactor device (U-CUP, Cellec Biotek AG) according to the manufacturer's instructions. Superficial velocity of 4.7 ml/min was applied for the first 18 h to allow initial cell adhesion, then changed to 0.47 ml/min until 28 days. In the meantime, 1×10^6^ cells were aliquoted, collected and stored at −20°C in order to be used as DNA or RNA reference.

After a total of 3 days for cell expansion, proliferation medium was replaced by differentiation medium by adding 100 µM ascorbic acid 2-phosphate (Sigma-Aldrich), 10 mM β-glycerophosphate (BGP), 100 nM dexamethasone (Millipore) and 10 nM 1,25-Dihydroxyvitamin D3 (Sigma-Aldrich) for MG-63 cells, or by adding 10 mM BGP and 10 nM 1,25-Dihydroxyvitamin D3 for hBMSC cells. Ten mM BGP was used according to established protocols (Zuk et al., 2002), it has to be noted, however, that free phosphate ion concentration might vary during cultivation (Schäck et al., 2013). The differentiation medium was replaced twice a week. After 18 h, 3, 7, 14 and 28 days of culture, the cell constructs were used for assays of cell viability and proliferation, DNA content, cell adherence, and osteoblast differentiation as described in the following paragraphs (*n*=4 for each assay). After cutting each scaffold in two, three or four parts with a sterilized scalpel, each piece of scaffold was weighed and characterized to determine cell seeding efficiency, cell proliferation and differentiation.

### Cell viability

To observe cell viability and distribution on the scaffold, the MTT [3-(4,5-dimethylthiazol-2-yl)-2,5-diphenyltetrazolium bromide] assay was performed. The MTT assay is a measure of metabolic activity. According to each time point designed for the MTT assay (*n*=4 for each time point), scaffolds were taken out from the 24-multiwell plate (static condition) or from the U-CUP perfusion bioreactor (dynamic condition). Briefly, each scaffold was transferred into a new 24-multiwell plate and incubated into 1 ml of complete medium mixed with 100 µl of MTT reagent (12 mM). Following 3 h of incubation at 37°C and 5% CO_2_, each scaffold was rinsed and stored in 1 ml of 1× PBS and observed using an inverted microscope (Zeiss Axiovert 40 CFL).

### Cell proliferation assay

The number of MG-63 or hBMSC cells that attached and grew on the scaffold was determined by quantifying the total DNA content. Briefly, each sample was incubated in 500 µl of phosphate buffered extraction solution (PpK) at 56°C for 16 h. To avoid any DNA-ceramic binding, the PpK solution was obtained by supplementing a proteinase K solution (pK; prepared by adding proteinase K 1 mg/ml, pepstatin A 19 µg/ml, EDTA 1 mM, iodoacetamide 1 mM, TRIS 50 mM to distilled water; Sigma-Aldrich) with potassium phosphate salts (HK_2_PO_4_ and H_2_KPO_4_; Fluka, Sigma-Aldrich) (Piccinini et al., 2010). DNA quantification was performed using the Quant-iT™ PicoGreen^®^ dsDNA Assay kit (Invitrogen) following the manufacturer's protocol. After incubation, the specimens were centrifuged at 10,000 ***g***, 4°C for 5 min. 5 µl of supernatant from each sample was added to 195 µl of PicoGreen^®^ reagent working solution in a 96-well black flat bottom plate. The analyses were carried out by measuring the fluorescence with a FlexStation™ 3 Microplate Reader (Molecular devices, USA), and the data were analysed using SoftMax Pro software. Excitation and emission wavelengths were 485 nm and 535 nm, respectively. A calibration curve was prepared in parallel by diluting a lambda standard DNA (1 µg/ml) to different concentrations (0, 50, 200, 600 and 1000 ng/ml). From the fluorescence obtained for the samples, the 0-value was subtracted and then divided through the slope of the standard curve (R^2^=0.999). Each sample and standard was measured in triplicate. The total number of cells per scaffold was determined by dividing the total DNA amount by the DNA amount per cell. The latter was obtained by keeping 2 million of cells separate (as a reference) and determination of the DNA amount for these non-seeded cells.

### ALP assay

ALP activity was quantified using an enzymatic assay based on the hydrolysis of *p*-nitrophenyl phosphate (pNP-PO_4,_ colourless) to *p*-nitrophenol (pNP, yellow) (Kim et al., 2012). ALP activity was quantified by the SensoLyte^®^ pNPP Alkaline Phosphatase Assay Kit (Anaspec). Working solutions were prepared according to the manufacturer's protocol. Each scaffold was washed twice with 1× assay buffer, crushed into small pieces and incubated in 500 µl lysis buffer [0.2% (v/v) Triton 100x] at 4°C for 10 min. After incubation, the samples were centrifuged at 2500 ***g***, 4°C for 5 min to remove scaffold debris. 50 µl of supernatant from each sample containing alkaline phosphatase was mixed with 50 µl of pNPP substrate solution into 96-well plates in triplicates. After 30 min of incubation at room temperature (RT), the reaction was stopped by adding 50 µl of stop solution, and the colorimetric determination of the product was performed at 405 nm using FlexStation™ 3 Microplate Reader. Twofold serial dilutions from 0.2 µg/ml ALP standard were made to prepare a calibration curve. The data were analysed using SoftMax Pro software. Results were normalized to the total cell number which was determined by the PicoGreen^®^ assay. ALP activity was expressed in ng p-nitrophenol produced/30 min/cell.

### Scanning electron microscopy (SEM)

To evaluate cell adhesion and ECM production/maturation, SEM analysis was performed. Cell-seeded scaffolds were washed in PBS 1× and fixed overnight in 2.5% (v/v) glutaraldehyde solution (Sigma-Aldrich) at 4°C. Dehydration was achieved by sequential immersion in serial diluted ethanol solutions of 30, 50, 70, 80, 90, 95 and 100% (v/v) for 15 min each, followed by critical point drying (AUTOSAMDRI-815, Tousimis). Finally, samples were sputter-coated with gold (thickness of 30 nm) using a Leica EM AC600, and examined using a NOVA NANOSEM 230 scanning electron microscope (Pharmazentrum ZMB, University of Basel). HA scaffolds without cells were used as negative control.

### Gene expression analysis using qPCR

After 3, 7, 14 and 28 days of culture, total RNA was isolated from the samples using TRIzol (Life Technologies) according to the manufacturer's instructions.

#### RNA extraction

Samples were crushed and 500 µl of cold TRIzol were added to each sample. 100 µl of chloroform were added to the homogenate, and incubated at RT for 3 min. After centrifugation at 12,000 ***g*** for 15 min at 4°C, the upper aqueous phase containing the RNA was collected and precipitated with 250 µl of isopropanol. To facilitate the precipitation, 2 µl of glycogen (Life Technologies) were added. Samples were incubated at RT for 10 min, and centrifuged at 12,000 ***g*** for 10 min at 4°C. The washing step was done with 75% ethanol, and the pellet was air-dried for 10 min before being resuspended in RNase-free water. The concentration and purity of each sample were assessed by the absorbance at 260 nm and by the A_260_/A_280_ ratio, respectively. RNA amounts were assessed with a NanoDrop^®^ 2000C (Thermo Fisher Scientific), and data were analysed with NanoDrop 2000/2000C software. All samples were diluted to obtain final concentrations of 10 ng/µl.

#### cDNA synthesis

1 µl of random primers (Promega) was added to 19 µl of RNA (10 ng/µl) and incubated at 70°C for 10 min to straighten the RNA. 10 µl of reaction mix was prepared per sample and added to preincubated RNA [0.5 µl Reverse transcriptase Superscript III RT 200 U/µl, 0.8 nM dNTP mix, 6 µl 5× first-strand buffer, 1 µl DTT and RNase-free water (Life Technologies)]. The mixture was treated as follows: 25°C for 10 min, 48°C for 30 min and 95°C for 5 min (Biometra, T3000 Thermocycler).

Reverse transcriptase polymerase chain reaction (RT-qPCR) assays were performed to determine the level of mRNA transcripts of the following genes of interest: ALP, osteocalcin (OC), collagen type I (COL1A1), vascular endothelial growth factor (VEGF), cluster of differentiation 31 (CD31), endothelial nitric oxide synthase (eNOS) and glyceraldehyde-3-phosphate dehydrogenase (GAPDH) as reference gene. Table 1 shows the sequences of the oligonucleotides that were used as PCR primers. All primers were purchased from Microsynth AG and reconstituted with Nuclease-free water to obtain 100 µM stock solutions.

**Table 1.**
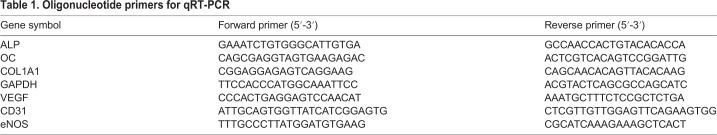
**Oligonucleotide primers for qRT-PCR**

Briefly, the reaction volume (20 µl) included 12.5 µl FastStart SYBR Green Master Mix (Roche), 2.5 µl diluted cDNA (10 ng/µl), 2 µl Primers mix (forward and reverse, 0.375 µM each) and 3 µl Nuclease-free water. After initial denaturation at 94°C for 5 min, the target genes were amplified with 45 quantification cycles (Cq) of denaturation at 94°C for 20 s and annealing at 58°C for 60 s. Afterwards, a dissociation cycle was performed from 50°C to 99°C (1°C every 5 s for each quantification cycle). The melting curve for each amplicon was performed to ensure the assay specificity validation. Real-time PCR reactions were carried out by rotor-Gene^®^ Q and Corbett devices (Qiagen). Data were analysed with Rotor-Gene 6000 Series Software and the levels of RNA expression were calculated according to the 2^−ΔΔCq^ method (Livak and Schmittgen, 2001). The expression level of each target gene was normalized to GAPDH as reference gene. The fold changes were calculated using Eqns 1-3 below:
(1)
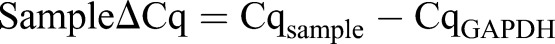

(2)
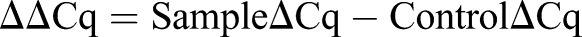

(3)
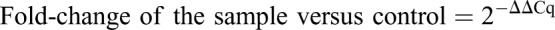


Each sample was assessed in three technical replicates for each gene of interest.

### Immunohistochemistry and histological staining

Immunohistochemistry analyses were performed to characterize cellular morphology, distribution and ECM maturation on HA scaffolds. Cell morphology was investigated by examining the F-actin cytoskeleton fluorescently stained with Texas Red [Texas Red-X-Phalloïdin, and the nucleus stained with DAPI (Invitrogen)]. To detect ECM maturation, a MAb Mouse IgG1 anti-Human Osteocalcin (R&D) was used as primary antibody, and a Goat anti-Mouse IgG (H+L) antibody labelled with Alexa Fluor^®^ 488 (Invitrogen) was used as a secondary antibody. Samples were washed with 1× PBS for 5 min, fixed with 4% (w/v) paraformaldehyde for 10 min, and rinsed three times with 1× PBS for 3 min each. Permeabilization was carried out using 0.1% Triton X-100 for 5 min. After washing three times with 1× PBS, samples were blocked with 5% (w/v) Bovine Serum Albumin solution (BSA) for 1 h in order to remove unspecific background. The samples were then incubated with Texas Red-X Phalloïdin diluted in 1% (w/v) BSA solution with a ratio of 1:500 (13.2 nM) at RT for 20 min. Samples were rinsed twice with 1% BSA solution for 3 min each, and incubated with 2.5 µg/ml osteocalcin primary antibody at RT for 2 h. After extensive washing steps, samples were incubated with 5 µg/ml secondary antibody at RT for 45 min. After further washing steps, samples were incubated with 0.95 µM DAPI at RT for 2 min followed by a final washing step with 1× PBS. Pictures of entire scaffolds on a glass slide were taken using an Olympus Laser Confocal Scanning Microscope FV1000D spectral type.

For the histological staining, constructs retrieved at 14 and 28 days of cell seeding were fixed overnight in 4% paraformaldehyde at 4°C. After fixation, samples were decalcified by incubation in a solution with 7 % (w/v) EDTA (7×10^4^ µg/ml) and 10% (w/v) sucrose (10^5^ µg/ml) at 37°C, 5% CO_2_ on an orbital shaker for 8-10 days. The solution was changed every 2 days and the hardness of the scaffold was checked daily. After washing in 1× PBS, samples were paraffin-embedded (TPC 15^DUO^, Medite TBS88 Paraffin embedding system cool unit, Switzerland) and sectioned (7 µm thick) by means of a microtome (Zeiss HYRAX M55). Paraffin sections were deparaffinized, hydrated and stained with Hematoxylin and Eosin (H&E) for nuclei and cytoplasm, respectively (Medite Tissue Stainer COT 20). Histology samples were observed under an Olympus CKX41 inverted microscope (*n*=4 for each condition).

### Mechanical testing

Scaffolds immersed in solution (wet condition) were tested by nanoindentation using the Piuma nanoindenter (Optics11) (*n*=3–4). The Young's Modulus was calculated using the data in the unloading curve with nine unique spots on each scaffold, making a slope estimate of all data points between 65 and 85% of the maximum load, and using the Oliver & Pharr model to calculate the efficiency ‘E’. The probe used was 173 N/m and 30.5 µm. For the cell-seeded scaffolds, differentiation induction medium was used.

In addition, standard compression tests were performed with a Hydropulser LFV-5-PA/ECD (Walter and Bai AG, Switzerland) under wet conditions to compare the elastic modulus (MPa) for cell-free and cell-seeded scaffolds (*n*=3).

### Devitalization/decellularization

After ECM deposition, samples were devitalized to obtain 3D-printed porous HA scaffolds coated by ECM without cells. Briefly, samples underwent three freeze and thaw (F/T) cycles in liquid nitrogen and 37°C water bath (10 min each), respectively. Scaffolds were rinsed in sterile PBS after each thaw step as well as in double-distilled water after the second thaw in order to hypotonically lyse remaining cells. To decellularize HA scaffolds (to eliminate cellular debris), a perfusion-based washing step was added subsequent to the F/T. The constructs were placed into the bioreactor system and perfused at 0.47 ml/min in PBS for 30 min at room temperature. To verify if the perfusion washing step was effectively removing cellular debris, a DAPI staining was performed and visualized using an inverted microscope.

### CAM assay and MRI assessment

No IACUC approval is necessary when performing experiments in chicken embryos until embryonic day 14 (ED 14) according to Swiss animal care guidelines (TSchV, Art. 112). Fertilized Lohman white LSL chicken eggs (Animalco AG, Staufen AG, Switzerland) were pre-incubated for 3 days at 37°C at a rotation speed of 360°/12 h. On ED 3 the eggs were processed for *in ovo* cultivation, which requires the opening of the shell with a drill (Dremel^®^, Conrad Electronic AG, Wollerau SZ, Switzerland). 2 ml of albumen was always removed with a syringe to increase the empty space under the top of the egg shell. The eggs were stabilized in 60 mm Petri dishes (Greiner Bio-One GmbH, Frickenhausen, Germany) and the created holes of the shells were covered with another 60 mm Petri dish that was fixed with a tape before incubating the eggs at 37°C. On ED 7, the decellularized HA scaffolds coated with either ECM produced from cells in proliferation medium or in osteogenic medium were placed on the CAM in the middle of silicon rings that ensure a flat surface during their incubation period of 7 days (*n*=4 for each group with ECM coating). As a control, ECM-free HA scaffolds were also placed on top of the CAM (*n*=6).

Vascularization of the scaffolds by capillaries of the chicken embryo's CAM was studied on ED 14 using MRI as previously described (Kivrak Pfiffner et al., 2014). The eggs were placed onto a custom-built sliding bed and enveloped by warm water tubing to maintain the temperature of the chicken embryo in a physiological range. To prevent motion, the chicken embryo was sedated with five drops of 1:100 M ketamine (Ketasol-100, Dr E. Graeub AG, Bern BE, Switzerland) dripped onto the CAM surface. MRI was performed with a 4.7 T/16 cm Bruker PharmaScan small animal scanner (Bruker BioSpin MRI GmbH, Ettlingen, Germany) equipped with an actively decoupled two-coil system consisting of a 72 mm bird cage resonator for excitation and a 20 mm single loop surface coil for reception. T1-weighted MR images were acquired with a RARE sequence of variable TR and TE for quantitative T1 and T2 mapping. T1 maps were acquired in the samples before and after intravenous injection of 0.05 M Gd-DOTA MRI contrast agent (Dotarem^®^, Guerbet AG, Zuerich ZH, Switzerland). The time between Gd-DOTA injection and T1 mapping was kept constant at 25 min. T1 relaxation times were determined in three layers of interest: at the interface of the scaffold with the CAM (i.e. lower part), in the middle part of the scaffold, and finally at the surface of the scaffold (i.e. upper part); for each layer, three ROIs were assessed, resulting in nine ROIs per scaffold. Perfusion capacity in these ROIs was assessed through changes in the longitudinal relaxation rate ΔR1 before and after injection of Gd-DOTA, as the relaxation rate changes with the amount of gadolinium present in the CAM.

### Statistics

The data was analysed with StatView 5.0.1 software. One-way statistical ANOVA was conducted to test the significance of differences between cell numbers at different days, ALP activities or manifold inductions of gene expression. Pairwise comparison probabilities (p) were calculated using the Tukey–Kramer HSD post hoc test to evaluate differences between the groups. *P*-values<0.0001 were considered significant, except for the perfusion capacity as assessed with MRI, where *P*<0.05 was considered significant and *P*-values were given for the significantly different groups. Values are expressed as means±standard deviations.
